# Resistance is reality: findings from the first Ukrainian cumulative antibiogram

**DOI:** 10.1093/jacamr/dlae156

**Published:** 2024-10-09

**Authors:** Arkadii Vodianyk, Oksana Holovnia, Eugene Diomin, Alyssa R Letourneau, Mark C Poznansky, Erica S Shenoy, Sarah E Turbett

**Affiliations:** Service Delivery Department, WHO Country Office for Ukraine, Kyiv, Ukraine; Department of Bacteriology, National Children’s Specialized Hospital Okhmatdyt, Kyiv, Ukraine; Service Delivery Department, WHO Country Office for Ukraine, Kyiv, Ukraine; Division of Infectious Diseases, Massachusetts General Hospital, Boston, MA, USA; Harvard Medical School, Boston, MA, USA; Division of Infectious Diseases, Massachusetts General Hospital, Boston, MA, USA; Harvard Medical School, Boston, MA, USA; Heal Ukraine Group, Boston, MA, USA; Division of Infectious Diseases, Massachusetts General Hospital, Boston, MA, USA; Harvard Medical School, Boston, MA, USA; Infection Control, Massachusetts General Hospital and Mass General Brigham, Boston, MA, USA; Division of Infectious Diseases, Massachusetts General Hospital, Boston, MA, USA; Harvard Medical School, Boston, MA, USA; Department of Pathology, Massachusetts General Hospital, Boston, MA, USA

## Abstract

**Background:**

Antimicrobial resistance is a global health threat resulting in significant morbidity and mortality worldwide. Until recently, in Ukraine, cumulative antibiograms (CuAbgms) have never been available.

**Objectives:**

To describe the first CuAbgm developed in Ukraine.

**Methods:**

We developed a CuAbgm for the Okhmatdyt National Specialized Children’s Hospital using data from WHONET. Antimicrobial susceptibility testing was performed per EUCAST guidelines. The CuAbgm was developed using guidance from CLSI.

**Results:**

For *Escherichia coli*, 66% and 69% of isolates were susceptible to ceftazidime and ceftriaxone, respectively, and 99% were susceptible to meropenem. For *Klebsiella pneumoniae*, 26% and 27% of isolates were susceptible to ceftazidime and ceftriaxone, respectively, and only 59% were susceptible to meropenem. Of the carbapenem-resistant *K. pneumoniae* isolates that underwent additional susceptibility testing, only 38% were susceptible to ceftazidime/avibactam. For *Pseudomonas aeruginosa*, only 53% were susceptible to meropenem. Of those that were resistant to meropenem and underwent additional susceptibility testing, only 12% were susceptible to ceftazidime/avibactam. Similarly, for *Acinetobacter* spp., only 37% of isolates were susceptible to meropenem. Susceptibility to ampicillin/sulbactam was also low at 45%. The oxacillin susceptibility rate for *Staphylococcus aureus* was 99%.

**Conclusions:**

In this first-ever CuAbgm developed in Ukraine, high levels of resistance were demonstrated among Gram-negative bacteria. CuAbgms should be prioritized in laboratories in Ukraine to guide empirical antimicrobial therapy, infection control and antimicrobial stewardship policies. This is of heightened relevance during wartime, when there is a need for healthcare systems to treat complex and infected penetrating and blast-related injuries.

## Introduction

Antimicrobial resistance (AMR) is a global health threat leading to significant morbidity and mortality worldwide.^1^ It has been estimated that 1.27 million deaths can be directly attributed to AMR infections annually, with up to 4.95 million deaths associated with AMR infection per year.^2^ AMR prevalence is geographically variable;^2^ identifying trends in local resistance patterns is useful to guide empirical antimicrobial therapy, improve antimicrobial stewardship efforts and enhance infection control prevention strategies.^3^ Cumulative antibiograms (CuAbgms), which provide antimicrobial susceptibility profiles for common microorganisms, are usually developed by healthcare systems to identify specific AMR trends inherent to their population to help guide therapeutic, stewardship and infection control practices.^3^ Although CuAbgms are frequently available in healthcare facilities across the globe, in some developing countries, such as Ukraine, antibiograms are not routinely available, largely due to a lack of standardized laboratory information systems needed for their development. Therefore, data on local resistance patterns in Ukraine are limited, preventing the implementation of rational and effective antimicrobial stewardship and infection control strategies to interrupt nosocomial transmission.

In 2019, with the support of WHO, a national action plan to combat AMR was implemented by the Ukrainian government, mandating hospitals to improve infection control and prevention practices, develop antimicrobial stewardship programmes, and limit unnecessary antimicrobial use. As a part of this initiative, Okhmatdyt National Specialized Children’s Hospital, a paediatric referral hospital in Kyiv, Ukraine, was given support to develop its first hospital-specific CuAbgm. Here, we describe the results of this first institution-specific antibiogram.

## Methods

We developed a CuAbgm for the Okhmatdyt National Specialized Children’s Hospital, which is the largest paediatric hospital in Ukraine. It is a 720 bed facility that has 20 000 admissions and performs 9000 procedures annually. Specialized procedures include bone marrow, liver and kidney transplantation. The hospital also provides specialty care to children with penetrating and blast-related injuries. For 2022 (the year the Russian invasion of Ukraine began), the hospital remained fully operational, with a similar number of patient admissions compared with previous years and included specialty care for patients with war-related injuries.

### Data extraction

To develop the CuAbgm, we reviewed clinical laboratory data from the Okhmatdyt Clinical Microbiology Laboratory using WHONET 2023, which is a free software program designed to analyse antimicrobial susceptibility testing (AST) data for antimicrobial resistance surveillance.^4^ This program uses BacLink software to import bacterial isolate information in a standardized format and provides data analysis tools for CuAbgm generation.^4^ Data importation into WHONET was performed by a limited set of qualified staff to ensure data accuracy and integrity.

### Bacterial isolate selection for inclusion in the CuAbgm

Using the WHONET software, we performed bacterial isolate selection using guidance from CLSI.^5^ Specifically, we identified bacterial isolates detected from positive blood cultures, lower respiratory tract and urinary specimens that underwent routine AST for diagnostic purposes from January 2022 to December 2022. Bacterial identification was performed using MALDI-TOF (VITEK^®^ MS, bioMérieux^®^, Marcy-l’Étoile, France). AST was performed using internally validated protocols in accordance with EUCAST or CLSI guidelines.^6,7^ ESBL production was confirmed for Enterobacterales that tested resistant to ceftazidime and/or ceftriaxone per EUCAST guidelines.^6^ Carbapenemase detection, including phenotypic testing that can differentiate between MBLs and selected serine-β-lactamases, was performed for Enterobacterales per EUCAST recommendations, but testing results were not readily available for CuAbgm incorporation at the time of data generation.^6^

Once the initial bacterial isolates and AST profiles were identified, we curated the dataset to only include the first isolate of a given species from a patient per the analysis period, regardless of the sample type and antimicrobial susceptibility profile. Repeat isolates from the same patient were excluded. All data processing was done within the WHONET software. Additionally, we only included antimicrobial agents that were routinely tested against the selected isolates, ensuring that each antimicrobial included was appropriate for the species. For colistin and ceftazidime/avibactam, AST was only performed routinely for all clinically significant isolates (from positive blood cultures or isolates from critically ill patients or organ recipients) or after confirmation of carbapenem resistance. AST profiles for the reported organism/antimicrobial combinations reflect testing from only a subset of these organisms identified in the laboratory. To guarantee statistical validity of the susceptibility estimates, when possible, only species with AST data for at least 30 isolates were included in the analysis.

### Data analysis

Using the WHONET software, we determined the percent susceptible (%S) and the percent ‘susceptible, increased exposure’ rates (%I) for each organism/antimicrobial combination per EUCAST recommendations.^8^ For a given organism, a susceptible result reflects a high likelihood of therapeutic success when a standard dosing regimen is used, whereas a susceptible, increased exposure result reflects a high likelihood of therapeutic success at certain sites of infection (due to higher concentrations of the antimicrobial at that site) or through higher antimicrobial dosing regimens.^8^ We denoted expected resistant phenotypes (intrinsic resistance) for selected organism/antimicrobial combinations as ‘R’ per EUCAST recommendations.^9^ Organism/antimicrobial combinations for which there were insufficient data or no clinical breakpoints were marked with an ‘x’; those combinations that were not routinely tested were marked with an ‘—’.

### CuAbgm comparison with Ukraine national surveillance data

To illustrate the importance of generating local, institution-specific antimicrobial susceptibility data to drive treatment, stewardship and infection control strategies, we compared the Okhmatdyt National Specialized Children’s Hospital CuAbgm to Ukraine national cumulative antimicrobial susceptibility data obtained from the Central Asian and European Surveillance of Antimicrobial Resistance Network (CAESAR).^10^ CAESAR is a network of national AMR surveillance systems designed to provide country-level antimicrobial susceptibility data for the WHO European Regions that are not part of the EU.^10^ The results presented in the CAESAR report are based on AMR data from invasive isolates (blood and CSF), reported to the CAESAR network and the European Antimicrobial Resistance Surveillance Network (EARS-Net) in 2022. In total, 16 countries reported data to CAESAR, while 29 countries, including those in the EU and 2 from the European Economic Area (EEA) (Iceland and Norway), reported data to EARS-Net.

## Results

### CuAbgm for selected Gram-positive bacteria

The CuAbgm for selected Gram-positive bacteria is summarized in Table 1. For *Staphylococcus aureus*, we found high susceptibility rates to oxacillin (99%), vancomycin (100%) and linezolid (100%). Penicillin susceptibility rates were also relatively high (41%). For the enterococci, we identified large differences in ampicillin and vancomycin susceptibility rates between *Enterococcus faecalis* and *Enterococcus faecium*, with at least 98% of *E. faecalis* isolates susceptible to ampicillin and vancomycin, but only 7% and 57% of *E. faecium* isolates susceptible to these drugs, respectively. Both species were uniformly susceptible to linezolid (100%).

**Table 1.** Cumulative antibiogram for selected Gram-positive bacteria

Bacterium	No. of strains	% S (%I)												
		Penicillin	Ampicillin	Oxacillin	Gentamicin	Amikacin	Tobramycin	Erythromycin	Clindamycin	Vancomycin	Ciprofloxacin	Moxifloxacin	Linezolid	Rifampicin^a^
*S. aureus*	138	41	x	99	98^b^	99^b^	97^b^	79	83^c^	100^d^	98^b^	96	100	100
*E. faecalis*	159	x	(98)	—	77^e^	R	R	R	R	99	76^f^	—	100	—
*E. faecium*	72	x	(7)	—	54^e^	R	R	R	R	57	19^f^	—	100	—

%S, percent susceptible, standard dosing regimen; %I, percent susceptible, increased exposure; R, expected resistance phenotype; x, drug not routinely tested; —, clinical breakpoints unavailable or insufficient data for successful treatment.

^a^Rifampicin should not be used alone for therapy.

^b^For systemic infections due to *S. aureus*, aminoglycosides and ciprofloxacin should not be used alone for therapy.

^c^Strains with a positive D-test were excluded.

^d^%S was determined for only 47 strains.

^e^%S reflects isolates that tested negative for the presence of aminoglycoside-modifying enzymes (high-level aminoglycoside resistance). Due to low-level natural resistance, aminoglycosides should not be used for monotherapy even if high-level resistance is not detected.

^f^For treatment of uncomplicated urinary tract infection only.

### CuAbgm for selected Gram-negative bacteria

The CuAbgms for enteric and non-enteric Gram-negative bacteria are summarized in Tables 2 and 3. For *Escherichia coli*, 66% and 69% of isolates were susceptible to ceftazidime and ceftriaxone, respectively, with ESBL production confirmed for 28% of isolates. Nearly all isolates (99%) were susceptible to meropenem. Of the ESBL-producing *E. coli* isolates that underwent colistin susceptibility testing (*n* = 25), 100% had MICs of ≤2 mg/L. In contrast, for *Klebsiella pneumoniae*, 26% and 27% of isolates were susceptible to ceftazidime and ceftriaxone, respectively, with ESBL production confirmed for 31% of isolates. More than half (59%) of isolates were susceptible to meropenem. Of the carbapenem-resistant *K. pneumoniae* isolates that underwent additional susceptibility testing (*n* = 60), 59% had colistin MICs of ≤2 mg/L and only 38% were susceptible to ceftazidime/avibactam (data not shown).

**Table 2.** Cumulative antibiogram for selected Enterobacterales

Bacterium	No. of strains	% S (%I)													
		Ampicillin	Amoxicillin/clavulanate	Piperacillin/tazobactam	Cefuroxime^a^	Ceftazidime	Ceftriaxone	Cefepime	Meropenem	Gentamicin^b^	Amikacin^b^	Tobramycin^b^	Ciprofloxacin	Trimethoprim/sulfamethoxazole	Colistin^c^
*E. coli*	144	37	65	83	72	(66)	(69)	(67)	(99)	82	84	77	(67)	(52)^d^	x
ESBL *E. coli*	41	0	20	56	0	(0)	(0)	(5)	(100)	61	70	54	(59)	(38)^d^	100^e^
*K. pneumoniae*	172	R	28	44	37	(26)	(27)	(23)	(59)	51	47	40	(34)	(34)	x
ESBL *K. pneumoniae*	53	R	23	60	0	(0)	(0)	(0)	(100)	59	60	45	(42)	(23)^f^	78
Carbapenem-resistant *K. pneumoniae*	60	R	0	0	0	(0)	(0)	(0)	(0)	18	8	3	(6)	(21)	59

%S, percent susceptible, standard dosing regimen; %I, percent susceptible, increased exposure; R, expected resistance phenotype; x, drug not routinely tested.

^a^For the treatment of uncomplicated urinary tract infections only.

^b^For systemic infections, aminoglycosides should not be used alone for therapy. Testing only performed for clinically significant isolates (from positive blood cultures or isolates from critically ill patients or organ recipients) or after confirmation of carbapenem resistance.

^c^For systemic infections, colistin should not be used alone for therapy.

^d^%I was determined for only 27 strains.

^e^%S was determined for only 25 strains.

^f^%I was determined for only 9 strains.

**Table 3.** Cumulative antibiogram for *Acinetobacter* spp. and *P.aeruginosa*

Bacterium	No. of strains	% S (% I)											
		Ampicillin/sulbactam	Piperacillin/tazobactam	Ceftazidime	Cefepime	Meropenem	Gentamicin^a^	Tobramycin^a^	Ciprofloxacin	Trimethoprim/sulfamethoxazole	Aztreonam	Colistin^b^	Ceftazidime/avibactam^c^
*Acinetobacter* spp.	87	45^d^	—	—	—	(37)	37	32	(39)	(36)	R	100	—
Carbapenem-resistant *Acinetobacter* spp.	55	15	—	—	—	0	6	2	10	5	R	100	—
*P.aeruginosa*	120	R	(55)	(42)	(35)	(53)	—	49	(54)	R	(85)	100	55
Carbapenem-resistant *P. aeruginosa*	64	R	(19)	0	0	0	—	17	15	R	73	100	12

%S, percent susceptible, standard dosing regimen; %I, percent susceptible, increased exposure; R, expected resistance phenotype; x, drug not routinely tested; —, clinical breakpoints unavailable or insufficient data for successful treatment.

^a^For systemic infections, aminoglycosides should not be used alone for therapy.

^b^For systemic infections, colistin should not be used alone for therapy. Testing only performed for clinically significant isolates (from positive blood cultures or isolates from critically ill patients or organ recipients) or after confirmation of carbapenem resistance.

^c^Testing only performed for clinically significant isolates (from positive blood cultures or isolates from critically ill patients or organ recipients) or after confirmation of carbapenem resistance.

^d^%S determined using CLSI breakpoints.

For *Pseudomonas aeruginosa*, only 53% of isolates were susceptible to meropenem, 85% were susceptible to aztreonam and 100% had colistin MICs of ≤2 mg/L. Of those carbapenem-resistant isolates that underwent additional susceptibility testing (*n* = 64), only 12% were susceptible to ceftazidime/avibactam. Similarly, for *Acinetobacter* spp., only 37% of isolates were susceptible to meropenem. Susceptibility to ampicillin/sulbactam was also low at 45%. The susceptibility profile for carbapenem-resistant *Acinetobacter* spp. was particularly poor, with only colistin retaining reliable *in vitro* activity against this genus (Table 3).

### Comparison of CuAbgm with Ukraine national surveillance result

Comparison of susceptibility rates for selected antimicrobials between the Okhmatdyt National Specialized Children’s Hospital CuAbgm and CAESAR 2021^10^ is shown in Table 4. *For S. aureus*, our CuAbgm oxacillin susceptibility rate was higher than the Ukraine national surveillance result (99% versus 70%). In contrast, our CuAbgm *E. faecium* vancomycin susceptibility rate was lower (57%) compared with the Ukraine national surveillance results (93%). For *E. coli* and *K. pneumoniae*, our CuAbgm susceptibility rates for third-generation cephalosporins, carbapenems and quinolones were higher compared with Ukraine national surveillance results (Table 4). Finally, although both the Ukraine national surveillance data and our CuAbgm reveal low susceptibility rates to ceftazidime and piperacillin/tazobactam for *P. aeruginosa* and to carbapenems and quinolones for *P. aeruginosa* and *Acinetobacter* spp., all rates were higher for our CuAbgm compared with the CAESAR registry (Table 4).

**Table 4.** Comparison of susceptibility rates for selected antimicrobials between CAESAR 2021^10^ and the Okhmatdyt National Specialized Children’s Hospital CuAbgm

Bacterium	Antimicrobial	CAESAR 2021 %S	Okhmatdyt National Specialized Children’s Hospital CuAbgm %S
*S. aureus*	Oxacillin	70^a^	99
*E. faecalis*	Gentamicin^b^	66	77
*E. faecium*	Vancomycin	93	57
*E. coli*	Third-generation cephalosporins	43^c^	66–69^d^
	Carbapenems	90^e^	99^f^
	Quinolones	57^g^	67^h^
*K. pneumoniae*	Third-generation cephalosporins	10^c^	26–27^d^
	Carbapenems	36^e^	59^f^
	Quinolones	16^g^	34^h^
*P. aeruginosa*	Ceftazidime	19	42
	Piperacillin/tazobactam	25	55
	Quinolones	19^g^	54^h^
	Carbapenems	22^e^	53^f^
*Acinetobacter* spp.	Carbapenems	27^e^	37^f^
	Quinolones	22^i^	39^h^

%S, percent susceptible.

^a^%S based on cefoxitin, or if unavailable, oxacillin. If neither were available, molecular test results were used.

^b^%S reflects isolates that tested as negative for the presence of aminoglycoside-modifying enzymes (high-level aminoglycoside resistance).

^c^Cefotaxime, ceftriaxone or ceftazidime.

^d^Ceftazidime or ceftriaxone.

^e^Meropenem or imipenem.

^f^Meropenem only.

^g^Ciprofloxacin, levofloxacin or ofloxacin.

^h^Ciprofloxacin only.

^i^Ciprofloxacin or levofloxacin.

## Discussion

With the growing global prevalence of antimicrobial resistance, access to accurate and reliable antimicrobial susceptibility data is becoming increasingly important for hospitals to guide empirical antimicrobial therapy, optimize antimicrobial stewardship efforts and enhance infection control guidance.^11^ One method employed by hospitals to curate antimicrobial susceptibility data is the CuAbgm, which provides antimicrobial susceptibility profiles for common pathogens. Unfortunately, in Ukraine, CuAbgms have historically been unavailable, forcing clinicians to prescribe empirical antimicrobial therapy based on clinical experience and using data from CAESAR reports.^10^ Although CAESAR provides aggregate antimicrobial susceptibility from invasive bacterial isolates submitted by hospitals throughout Ukraine, these reports reflect previous years’ AST data, and are limited in terms of their scope and generalizability, preventing Ukrainian hospitals from developing data-driven institution-specific guidelines for empirical antimicrobial therapy, antimicrobial stewardship and infection control. Here, with support from the Ukrainian government and WHO, we report the development and findings of the first institution-specific CuAbgm in Ukraine and contrast its findings with Ukraine national surveillance data from CAESAR.

For the Gram-negative organisms, antimicrobial resistance was common, with low susceptibility rates identified for many antimicrobial categories. For *E. coli* and *K. pneumoniae*, resistance to third-generation cephalosporins was relatively common, with approximately one-third of *E. coli* and three-quarters of *K. pneumoniae* isolates expressing this phenotype. However, significant differences in carbapenem susceptibility were identified between these two species, with nearly all *E. coli* isolates retaining susceptibility to this drug class. This finding provides support for the empirical use of carbapenems for infections caused by this organism in our hospital. In contrast, carbapenem susceptibility of *K. pneumoniae* was lower, rendering this antibiotic class a suboptimal choice for empirical treatment of *K. pneumoniae* infections. This trend is consistent with those from the Ukraine national surveillance data, as well as major studies evaluating causes of neonatal sepsis in parts of Asia and Africa,^12^ and highlights the need for improved availability of expanded antimicrobial susceptibility testing for novel antimicrobials with activity against carbapenem-resistant Enterobacterales, including new β-lactam/β-lactamase inhibitors (BLBLIs) and the siderophore cephalosporin cefiderocol.

For a subset of our carbapenem-resistant *K. pneumoniae*, susceptibility testing for the new BLBLI ceftazidime/avibactam was performed; only 38% of these isolates were susceptible to this agent. Ceftazidime/avibactam has activity against serine-β-lactamases including KPC and OXA-48-like carbapenemases but no activity against MBLs such as NDM, VIM and IMP.^13^ Our low ceftazidime/avibactam susceptibility rate suggests a high prevalence of MBL-containing *K. pneumoniae* isolates within our tested population. A high prevalence of MBL-containing Enterobacterales has been described in refugees and war-wounded Ukrainians,^14^ which further supports this hypothesis. With such low susceptibility rates to ceftazidime/avibactam, there is limited utility of this agent for empirical treatment of carbapenem-resistant *K. pneumoniae* infections in our hospital without additional rapid resistance mechanism determination. Rapid molecular and phenotypic assays that can identify and differentiate among the common carbapenem resistance mechanisms are commercially available for use in clinical laboratories. In areas such as ours, where the mechanism of carbapenem resistance is genetically diverse, use of these assays could better inform the use of novel BLBLIs and cefiderocol in the empirical setting. In the absence of these tests, use of an empirical antibiotic regimen active against both serine and MBL carbapenemases is prudent if *K. pneumoniae* is identified.

Similar to CAESAR data for Ukraine, we identified uniformly low susceptibility rates to almost all tested antimicrobials for *Acinetobacter* spp. and *P. aeruginosa.* For *P. aeruginosa*, susceptibility to ceftazidime/avibactam was also low, with approximately half of all tested isolates and only 12% of carbapenem-resistant strains demonstrating susceptibility to this agent. These susceptibility rates are significantly lower than those reported for ceftazidime/avibactam and *P. aeruginosa* from other parts of the world, where resistance rates have been described to be anywhere from 1% to 18%.^15^ Numerous causes of ceftazidime/avibactam resistance in *P. aeruginosa* have been reported including porin mutations, overexpression of efflux pumps, and acquisition of MBL carbapenemases. The innumerable resistance mechanisms that can develop within this species highlight the challenges associated with treatment of this pathogen. Interestingly, aztreonam susceptibility remained high at 85%. Use of this drug in combination with other agents with activity against *P. aeruginosa* should be considered at our hospital.

For *Acinetobacter* spp., meropenem susceptibility was low (37%), which is consistent with rates reported in other eastern European countries, parts of Asia, and Africa.^12,16,17^ Susceptibility rates for ampicillin/sulbactam, which is considered a first-line treatment agent for carbapenem-resistant strains^18^ (due to the activity of the sulbactam component against PBP1 and PBP3^19^), was active against only 15% of our carbapenem-resistant isolates. Despite non-susceptibility, higher doses of ampicillin/sulbactam have been shown to retain some activity against this genus and this drug continues to be an important component of *Acinetobacter* directed therapy in combination with other agents.^20,21^ Newer antimicrobial agents, including the novel BLBLI sulbactam*-*durlobactam and cefiderocol, are also becoming increasingly available and have shown improved efficacy for treatment of carbapenem-resistant *Acinetobacter* spp. infections. Our data suggest utility of empirical use of these agents, if available, likely in combination with other agents, if *Acinetobacter* spp. is identified.^19,22^

Of all the antimicrobials tested for the Gram-negative bacteria, colistin maintained relatively high levels of *in vitro* activity, with 78% to 100% of ESBL-producing *K. pneumoniae* and *E. coli*, 59% of carbapenem-resistant *K. pneumoniae* and 100% of carbapenem-resistant *P. aeruginosa* and *Acinetobacter* spp. having colistin MICs of ≤2 mg/L. Colistin is a polymyxin antimicrobial that has emerged as a last-resort treatment option for drug-resistant Gram-negative infections, including selected members of the Enterobacterales order, *P. aeruginosa* and *Acinetobacter* spp. Use of this drug is often restricted to cases where other antimicrobial options are not available due to its limited clinical efficacy, unfavourable toxicity profile and challenges in performing accurate AST.^23,24^ Numerous studies have shown higher mortality rates with the use of this agent for treatment of serious infections caused by carbapenem-resistant Enterobacterales and carbapenem-resistant *Acinetobacter* spp., and greater risk of nephrotoxicity when used alone or in combination with other antimicrobials.^18^ In fact, these data, along with review of the pharmacokinetic and pharmacodynamic profiles of these drugs, led CLSI and EUCAST to eliminate the ‘susceptible’ interpretive category for polymyxins in an attempt to deter their use.^25^ Therefore, despite the high levels of *in vitro* activity, treatment of serious infections with colistin should be avoided whenever possible, and if used, should be combined with other agents with potential activity.

For the Gram-positive organisms, β-lactam susceptibility of *S. aureus* was high at our hospital, with almost half of isolates testing as susceptible to penicillin and nearly all of them testing as susceptible to oxacillin. These rates were similar to those reported in Nordic countries^26^ and were well above those reported by CAESAR for Ukraine,^10^ as well as those seen in Europe and the USA, where oxacillin susceptibility rates have been reported to be as low as 50% depending on the geographic location (https://sentry-mvp.jmilabs.com).^16^ Large differences were also seen between Ukraine national surveillance data and our CuAbgm for vancomycin for *E. faecium*, with slightly more than half of our isolates retaining susceptibility to this agent. Our vancomycin susceptibility results for *E. faecium* are similar to those reported for many other European countries but are lower than those reported in the USA (https://sentry-mvp.jmilabs.com).^16^ These differences in susceptibility profiles reflect the geographic variability of antimicrobial resistance and highlight the need for local CuAbgm development to guide empirical antimicrobial algorithms and hospital stewardship efforts. For example, based on our CuAbgm, empirical treatment regimens for presumed *S. aureus* infection could include only a β-lactam antibiotic with activity against MSSA given the high rates of oxacillin susceptibility. Broad-spectrum antimicrobials, such as vancomycin or linezolid, which have activity against oxacillin-resistant strains, could be reserved for cases where β-lactam antibiotics could not safely be used, which would reduce MDR organism (MDRO) selection pressure and prevent further AMR development.^27^ In contrast, although the Ukraine national surveillance data indicate that vancomycin is a reasonable treatment option for *E. faecium* infections, our CuAbgm suggests that treatment with this agent should be avoided given the low overall susceptibility rates. Instead, empirical therapy with a more broad-spectrum antimicrobial, such as linezolid, should be considered until formal susceptibility testing results are available.

Our study has strengths and limitations. The main strength is that the presented antibiogram was performed according to standard methodology,^5^ providing real-world data on common pathogens and their susceptibility to routine antimicrobials. Additionally, this project is part of a larger nationwide plan to combat AMR in Ukraine; our study provides a roadmap for further antibiogram development at other institutions to improve empirical antimicrobial selection. Limitations include a limited ability to determine resistance mechanisms, particularly for the carbapenem-resistant organisms. Although differentiation of carbapenemase production was performed in the laboratory, these test results were not readily available at the time of CuAbgm generation and were not used to guide ceftazidime/avibactam and colistin AST. This limited our ability to distinguish between different carbapenem resistance mechanisms for the purposes of CuAbgm generation. Other limitations include the reporting of only a limited selection of organisms and antimicrobial combinations. Finally, although we present the CuAbgm for the largest paediatric hospital in Ukraine, our data represent only a subset of AST data for the country, limiting the generalizability of our data to other hospitals, populations and the CAESAR network.

### Conclusions

In this first-ever CuAbgm developed in war-time Ukraine, high levels of antimicrobial resistance were demonstrated among Gram-negative bacteria. Rates of carbapenem-resistant *K. pneumoniae*, *P. aeruginosa* and *Acinetobacter* spp. were particularly high and additional testing for the new BLBLI ceftazidime/avibactam provided little additional benefit. These trends highlight the increasing problem of AMR globally and reinforce the need for robust infection control and antimicrobial stewardship policies. CuAbgm development should be prioritized in laboratories throughout Ukraine to help guide empirical antimicrobial therapy. This is of heightened relevance during wartime when there is a significant increase in the need to treat complex and infected penetrating and blast-related injuries.
